# Terrestrial Toxicity of Synthetic Gas‐to‐Liquid versus Crude Oil–Derived Drilling Fluids in Soil

**DOI:** 10.1002/etc.4658

**Published:** 2020-02-11

**Authors:** Lisa Arneson Westbrook, Darcy A. Chase, Joseph Mudge, Sarah A. Hughes, Delina Lyon, Meijun Dong, Deborah Carr, Todd A. Anderson

**Affiliations:** ^1^ Department of Environmental Toxicology Texas Tech University Lubbock Texas USA; ^2^ Shell Health–Americas, Shell Oil Houston Texas USA; ^3^ Department of Biological Sciences University of Alberta Edmonton Alberta Canada; ^4^ Department of Biological Sciences Texas Tech University Lubbock Texas USA

**Keywords:** Terrestrial toxicity, Gas‐to‐liquids, Nonaqueous base fluid, Sandy loam

## Abstract

Unlike most other conventional petroleum products that are derived from crude oil, gas‐to‐liquids (GTLs) are petroleum products that are synthesized from natural gas (methane). This process results in GTL products having no sulfur and low aromatic content, so they should have less impact on human health and the environment compared with crude oil–derived products. The GTLs have been registered for use as nonaqueous base fluids (NABFs) in drilling muds, which aid in the process of drilling wells for oil and gas extraction; it is through these uses and others that they enter terrestrial environments. The primary objective of the present study was to determine whether GTLs were less toxic to terrestrial soil biota than conventional NABFs used for land‐based drilling, such as diesel and low‐toxicity mineral oil (LTMO). A second objective was to understand the fate and impact of these fluids under more realistic soil and aging conditions of a common west Texas (USA) oil‐producing region (i.e., sandy loam soil with low organic matter and a hot arid climate). Acute terrestrial toxicity studies were conducted on the soft‐bodied terrestrial invertebrate earthworm (*Eisenia fetida*) along with 3 plant species—alfalfa (*Medicago stavia*), thickspike wheatgrass (*Elymus lanceolatus*), and fourwing saltbrush (*Atriplex canescens*). We also assessed changes in microbial community structure of the soils following additions of NABF. Overall, the GTL NABFs had lower toxicity compared with conventional NABFs like diesel and LTMO, as measured by invertebrate toxicity, plant seed germination, and impact on the microbial community. *Environ Toxicol Chem* 2020;39:721–730. © 2020 The Authors. *Environmental Toxicology and Chemistry* published by Wiley Periodicals, Inc. on behalf of SETAC.

## INTRODUCTION

Gas‐to‐liquids (GTLs) are hydrocarbon liquids that are synthesized from natural gas (methane), unlike most other conventional petroleum liquids that are derived from crude oil. The technology to produce GTL products was first developed in 1926 but was not considered economically feasible for commercial‐scale use. Today, it is economically feasible to produce GTLs, and they are entering the market in a variety of products including fuels, base oils, solvents, naphtha, waxes, and drilling base fluids. The GTL products tested in the present study are produced by mixing methane, steam, and oxygen in a reformer (a processing unit that uses high temperature and pressure with a catalyst) to produce a carbon monoxide/hydrogen mixture called syngas. Because GTL products are synthesized from methane, they contain only trace levels of sulfur and aromatic compounds compared with their crude oil–derived counterparts (Knottenbelt 2002). As a result, GTL products have lower mammalian toxicity (Boogaard et al. 2016).

The GTLs are being used as nonaqueous base fluids (NABFs) in drilling muds both onshore and offshore (International Association of Oil & Gas Producers 2016). Exposure of terrestrial organisms to NABFs can occur at multiple points in the life cycle of the fluid, including at drilling sites, in transit, and at disposal sites. During drilling, the drilling mud mixes with material from the borehole and carries it up to the surface. This mixture, termed drill cuttings, is often saturated with drilling mud and is treated using various physical separation methods to recover the drilling mud for recycling. The treated cuttings can be further processed to remove more of the drilling fluid, often measured as total petroleum hydrocarbon (TPH), prior to disposal.

Aging of NABFs in soil, involving sorption, volatilization, and biodegradation of the NABF, can result in changes in chemical composition and subsequently lower TPH concentration over time (Douglas 1996; Scow and Johnson 1996). One of the most economical ways to lower TPH in cuttings is through bioremediation methods like land farming and composting/biopiles, which take advantage of natural processes to lower any potential toxic effects of TPH in the terrestrial environment (Salanitro et al. 1997; Dorn and Salanitro 2000; Bento et al. 2005). The success of bioremediation is dependent on the chemical properties of the NABF, the climate, the physicochemical properties of the soil, and the soil microbial consortium (Scow and Johnson 1996; Ghazali et al. 2004). Lee et al. (2002) found that a synthetic linear paraffin (C_11_–C_17_), prepared using a Fischer–Tropsch process similar to the GTL products in the present study, biodegrades at a rate similar to other conventional NABFs.

Several studies have examined the terrestrial toxicity of oil‐contaminated soils (Salanitro et al. 1997; Dorn et al. 1998; Dorn and Salanitro 2000) using standard terrestrial toxicity test guidelines (Environment Canada 2007; Organisation for Economic Co‐operation and Development 2003). Lee et al. (2002) investigated the terrestrial toxicity of a variety of traditional (i.e., diesel and mineral oil) and synthetic NABFs. In that study, the researchers used standard agricultural species (lettuce [*Lactuca sativa*] and barley [*Hordeum vulgare*]) for germination tests and the earthworm (*Eisenia fetida*) for invertebrate tests. In all assays, synthetic NABFs were found to be less toxic than the diesel and mineral oil NABFs. Notably, this team performed their studies on a forest subsoil with higher organic carbon content (2.6–16%) than soils found in the active land‐based drilling areas of the Permian Basin in west Texas (USA), like the one used in the present study (sandy loams, typically <1% total organic carbon; Zobeck et al. 2006). Indeed, this is an important point of distinction because organic carbon is known to sorb organic compounds, thereby moderating terrestrial toxicity (Lanno et al. 2004; Reinecke et al. 2016) and improving biodegradation (Gainer et al. 2019a). For example, Gainer et al. (2019a) found higher toxicity of medium hydrocarbons to soil invertebrates in low organic matter Canadian field soils.

In the United States, diesel and low‐toxicity mineral oil are more commonly used NABFs for land‐based drilling. Therefore, the objective of the present study was to compare the relative terrestrial toxicity, in both freshly spiked and spiked soils that have been aged, of 2 different GTL NABFs (with carbon ranges of C_10_–C_22_ and C_11_–C_24_) along with 2 crude oil–derived NABFs (an ultra‐low‐sulfur diesel [diesel] and a low‐toxicity mineral oil [LTMO]). The soil we used was a sandy loam soil of low organic carbon content representative of active areas of drilling in west Texas. To determine whether and how the terrestrial toxicity of NABFs varied among organisms, we chose test species for their relevance to terrestrial environments and their acceptance as standard test organisms. Earthworms were used as an example of soft‐bodied invertebrates. Alfalfa (*Medicago stavia*), thickspike wheatgrass (*Elymus lanceolatus*), and fourwing saltbrush (*Atriplex canescens*) were all chosen for phytotoxicity studies as examples of a legume, a grass, and a halophyte (salt‐tolerant) species, respectively.

## MATERIALS AND METHODS

### Properties of test NABFs

Four NABFs were evaluated and compared for their terrestrial toxicity. Their properties are given in Table 1. The 2 GTL NABFs had a similar composition; GTL with a carbon chain length ranging from C_10_ to C_22_ was referred to as GTL_10–22_, and GTL with a carbon chain length ranging from C_11_ to C_24_ was referred to as GTL_11–24_. The other 2 NABFs included a LTMO (C_11_–C_15_) and an ultra‐low‐sulfur diesel (diesel, C_10_–C_24_). All test products were provided by Shell Technology Center (Houston, TX, USA).

**Table 1:** Chemical description and properties of nonaqueous base fluids

NABF	Description^a^
GTL C_10_–C_22_	Clear liquid, a synthetic GTL paraffin, aliphatic hydrocarbon mixture of C_10_–C_22_ alkanes, approximately 14% linear and 86% branched alkanes (predominantly mono‐methyl), approximately 0% weight of aromatics, flash pt. 85 °C, TPH profile alkanes: C_10_–C_22_
GTL C_11_–C_24_	Clear liquid, a synthetic GTL paraffin, middle distillates from Fisher–Tropsch method, hydrocarbon mixture of C_11_–C_24_ alkanes, approximately 13% linear and 87% branched alkanes (predominantly mono‐methyl), approximately 0% weight of aromatics, TPH profile alkanes: C_11_–C_24_
LTMO	A yellow liquid with approximately 0.2% weight of aromatics, flash pt. 78.8 °C, TPH profile alkanes: C_11_–C_15_
Diesel^b^	A red liquid with a diesel odor, contains sulfur (≤15 ppm), and 30–60% weight of aromatics, flash pt. 66.1 °C, TPH profile alkanes: C_10_–C_24_

^a^ Each description includes the alkane profiles used to identify and quantify the oil content for soils spiked with these oils.

^b^ Ultra‐low‐sulfur diesel.

NABF = nonaqueous base fluid; GTL = gas‐to‐liquid; TPH = total petroleum hydrocarbons; LTMO = low‐toxicity mineral oil.

### Properties of test soil

The test soil we used was an agricultural soil collected in Terry County (TX). This soil was chosen because it was readily available within regions of high drilling activity and because, as a natural soil, it would contain an established microbial community to maximize the potential for biological degradation. Soil characteristics were determined by A & L Midwest Laboratories (Omaha, NE, USA), a certified soil testing laboratory. The soil pH was 8.3 using a 2:1 water‐to‐soil ratio. The test soil texture, determined via the hydrometer method (Bouyoucos 1962), was 74% sand, 10% silt, and 16% clay; the organic carbon content was 1.3%. The low organic carbon content also allowed for toxicity testing under conditions of increased bioavailability.

### Addition of NABFs

Soils were spiked at 2% w/w (NABF/sandy loam soil) similar to previous studies investigating the biodegradability and toxicity of NABFs in drilling muds (Lee et al. 2002; Visser et al. 2002). Range‐finding tests performed in the present study confirmed that toxicity was present at a dosing of 2% w/w. For each NABF treatment, the NABF was dissolved into a minimal volume of acetone as a solvent vehicle, added to dry soil, and mechanically mixed for 30 min. After mixing, moisture was added using deionized (ASTM International type II) water to bring the soil to 26% of saturation (approximately field capacity moisture content). A vehicle (acetone) control soil and a true control (no treatment) were also included in our study, although the potential chemical interaction between acetone and the test chemicals is an uncertainty not accounted for by a vehicle control.

### Aging of test chemicals in soil

Because aging of contaminants in soils can result in a reduction of toxicity, soils in which chemicals were aged were tested, in addition to freshly spiked soils (Dorn and Salanitro 2000; Bento et al. 2005). Aged spiked soils were incubated covered in the dark (for consistency with other studies) at either a low temperature condition (10 °C) or a high temperature condition (30 °C) for 90 d. For each temperature, there were 6 types of soil treatment (control, vehicle control, GTL_10–22_, GTL_11–24_, LTMO, and diesel). Soil moisture was initially adjusted to 26% of saturation using ASTM International type II water. During the 90‐d aging process, the soil was checked biweekly to adjust moisture content and to mechanically mix the soil. We were unable to begin all toxicity tests concurrently, so once soils had completed the 90‐d aging period, they were stored at –20 °C until use (no more than 14 d).

Samples had been spiked initially at 2% w/w NABF, so the amount of oil/g of soil (dry wt) was expected to be approximately 20 000 µg in freshly spiked soils. We made no attempt to elucidate the mechanism(s) of NABF loss from the soil during the aging period, because that was not the purpose of our study; we assumed natural losses from soil and a reduction in bioavailability of all test materials over the 90 d at both 10 and 30 °C through volatilization, biodegradation, and sorption (Gainer et al. 2019a).

### Earthworm acute toxicity study

Acute toxicities of NABF treatments were assessed using the earthworm *Eisenia fetida*. A 14‐d earthworm survival test was conducted using freshly spiked and aged soils (incubated for 90 d at 10 or 30 °C). A previous study had shown that 14‐d survival tests are well suited for testing terrestrial soil toxicity to earthworms (Saterbak et al. 1999); we generally followed Organisation for Economic Co‐operation and Development (1984) test guideline 207 (including test validity based on survival of controls) with some minor exceptions (no artificial soil, mass of soil used, daily monitoring vs 7‐ and 14‐d monitoring, etc.). We chose this approach primarily based on our desire to use a real soil and to be able to determine a time‐to‐death metric. Cultures of earthworms were purchased from Yelm Earthworm and Casting Farm. Larger adults that possessed a well‐developed clitellum were used, and before the study commenced, earthworms were depurated for 24 h.

There were 6 NABF treatments (control, vehicle control, GTL_10–22_, GTL_11–24_, LTMO, and diesel) and 3 soil aging scenarios (freshly spiked and aged for 90 d at 10 or 30 °C), for a total of 18 treatments. There were 5 replicates for the controls and 6 replicates for each NABF treatment. Each replicate consisted of an amber glass jar (118 mL) filled with approximately 50 g of treated and moistened soil (control or 2% w/w of NABF; ~field capacity moisture content). Soil also had food (organic matter) added (~12% w/w mixture of cornmeal and used coffee grounds; Lee et al. 2002; Visser et al. 2002). Food was added because preliminary tests showed better earthworm survival in controls with food. After soil was added to the jars, a single worm was added to each jar, and then lids were placed loosely over the jars to allow for gas exchange but minimize moisture loss. Jars were incubated in the dark at 24 °C and monitored daily for 14 d. During this time, any additional moisture needed to maintain soil at field capacity (based on weight of the jar) was added as necessary.

### Plant germination studies

The potential phytotoxicity of NABFs was assessed using seed germination assays of 3 plant species: alfalfa, thickspike wheatgrass, and fourwing saltbrush. Seed germination is a sensitive and commonly used endpoint for testing the toxicity of hydrocarbon contaminants in soil (Saterbak et al. 1999). All seeds were purchased from Great Basin Seeds. As with the earthworm acute toxicity study, there were 18 treatments (treatment/aging combinations): 6 NABF treatments (control, vehicle control, GTL_10–22_, GTL_11–24_, LTMO, and diesel) and 3 soil aging scenarios (freshly spiked and aged for 90 d at 10 or 30 °C).

For both the alfalfa and wheatgrass assays, each of the 18 soil treatments had 5 replicates that were contained in a covered plastic Petri dish (35‐mm diameter) with 6 seeds and 10 g of moistened soil (control or treated 2% w/w of NABF; ~field capacity moisture content; Lee et al. 2002; Visser et al. 2002). Sample containers were monitored and adjusted for moisture daily (based on weight loss) and incubated in a growth chamber (24‐h dark cycle, 24 °C). Seeds were assessed for germination after 14 d.

For the saltbrush assay, initial pilot studies were performed to determine optimal germination conditions. Pilot studies yielded only 35% germination under optimal conditions for the saltbrush seeds; however, this percentage was consistent with previous data (Meyer 2008). It was also discovered that 21 d were needed for germination of some seeds, but that additional time beyond 21 d did not improve germination success. Due to the low germination proportion of saltbush, 100 seeds were used for each replicate, and there were 3 replicates for each of the 18 soil treatment and aging combinations. Replicates consisted of a polystyrene plate (110‐mm diameter), 90 g of moistened soil (control or treated 2% w/w of NABF; ~field capacity moisture content), 100 seeds, and parafilm covering each of the plates. These containers were monitored and adjusted for moisture daily (based on weight loss) and incubated in a growth chamber (24‐h dark cycle, 24 °C).

### Microbial community analysis

Microbial community composition was determined using 454 sequencing (Research and Testing Services) of total soil DNA extract using bacterial and fungal tag‐encoded FLX amplicon pyrosequencing (bTEFAP and fTEFAP). These methods are based on the Roche 454 FLX pyrosequencing platform (Dowd et al. 2008).

### Statistical analysis

The acute toxicities of NABFs to earthworms were evaluated using log rank tests for differences in survival over time between NABF and control treatments within each aging scenario. Optimal α levels that minimized the combination of type I and II error rates were used to evaluate the significance of the log rank tests (Mudge et al. 2012). The optimal significance levels were 0.083 and 0.102 for control versus NABF and NABF versus NABF tests, respectively, and were associated with 88 and 85% statistical power, for a critical effect size *w* = 0.75. This critical effect size approximately corresponds to a scenario of 90% control mortality with one random control death early in the experiment and 50% treatment group mortality, evenly distributed throughout the experiment.

For all germination studies, the proportion of seeds that germinated was calculated for each replicate within each treatment. Differences in germination proportions among treatments were evaluated using a one‐way randomization analysis of variance (ANOVA) for each aging scenario (Manly 2006). The optimal significance levels calculated to minimize type I and II errors for the randomization ANOVAs were 0.036 for the alfalfa and wheatgrass assays, and 0.125 for the saltbrush assay. These significance levels were associated with 97 and 90% statistical power to detect an effect size of *f* ^2^ = 1, which represents a variance among treatment groups that is as large as the within‐group variance. When randomization ANOVA detected significant among‐group variance, post hoc 2‐sample Monte Carlo tests were conducted to evaluate differences between treatments (Rothman 1990; Manly 2006). The optimal α levels for these tests were 0.105 for the alfalfa and wheatgrass assays, and 0.177 for the saltbrush assay, with corresponding statistical power of 91 and 87% to detect a 0.33 decline in germination proportion, respectively. All the statistical tests were performed using R Statistical Software, Ver 2.13.1.

### Analytical methods

Soil sample concentrations were confirmed using a quantitative method developed to analyze the n‐alkanes in GTLs. Samples were extracted with methylene chloride, filtered using a 0.2‐µm polytetrafluoroethylene filter, and then analyzed using an HP6890 gas chromatograph coupled with an HP5973 mass spectrometer (GC–MS) in the selected ion mode. Quantitation of hydrocarbons was accomplished using m/z = 85 (Frysinger et al. 2003).

The NABF content in soil samples over time (0–90 d) was quantified using a modified TPH method, Texas Natural Resource Conservation Commission (2001) method 1005, which included a calibration curve for each NABF on a GC–flame ionization detector (FID). The specific hydrocarbons that were abundant in each NABF were summed into the TPH response for the concentration of each NABF. The calibration curves were then created from these TPH responses of known concentrations and then used to calculate the NABF mass in each sample.

The NABF content in soil sample extracts was also tested using 2‐dimensional (2D) GC in laboratories at Shell Technology Centre Thornton, Shell Global Solutions (UK). In GC × GC, 2 independent GC separations are applied to the entire sample. The GC × GC analysis was performed on an Agilent Technologies 6890 GC device using an Optic IIPTV split mode injector. The data processing was performed and visualized with in‐house–developed software. The individual components found by GC × GC were grouped both on the basis of carbon number (C_5_–C_30_) and on the following chemical functionalities: normal paraffins, iso‐paraffins, mono‐naphthenics, di‐naphthenics, mono‐aromatics, naphthenic mono‐aromatics, di‐aromatics, naphthenic di‐aromatics, and tri‐aromatics.

## RESULTS

### NABF dissipation from soil

Analytical data (both GC–MS and 2D GC) were used to compare measured TPH concentrations with nominal at the beginning of the aging period. Measured NABF content ranged from 68 to 84% of nominal (combined data from both types of GC analysis). Dissipation of NABFs from sandy loam soil during the 90‐d aging period at either 10 or 30 °C was assessed from the GC–FID and 2D GC data. We observed loss of lighter alkanes from all test substances (at a faster rate at 30 °C than at 10 °C), as expected. Consequently, the LTMO, which consists mainly of C_12_ to C_14_ hydrocarbons, was rapidly lost relative to the other 3 NABFs, which contain high proportions of heavier hydrocarbons. Concentrations of C_18_ and heavier alkanes hardly changed during the 90‐d aging period, whereas the concentrations of the C_14_ and lighter alkanes were greatly reduced over the same period (Supplemental Data, Figure S1).

### Earthworm acute toxicity study

The control and vehicle control groups exhibited ≥80% survival over 14 d in each of the 3 aging condition experiments (freshly spiked, aged at 10 °C, aged at 30 °C). The maximum difference in mortality between control and vehicle control treatments (when it occurred) was one individual. Due to the consistently high survival in the control and vehicle control groups, the control and vehicle control data were pooled together in each aging treatment for all subsequent analyses.

**Figure 1:**
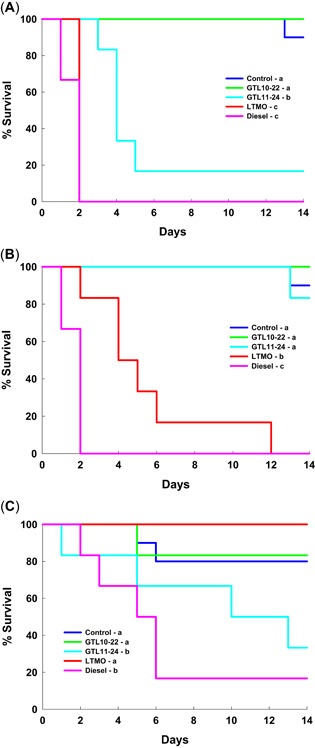
Acute toxicity of nonaqueous base fluids to the earthworm (*Eisenia fetida*). The colored lines represent cumulative percentage of survival at each day. Test substances were freshly spiked into sandy loams oil at 2% w/w and then used immediately (**A**) or incubated for 90 d at either 10 or 30 °C and then used in toxicity assays (**B** and **C**, respectively). Statistically significant differences in survival by day 14 between treatments are denoted in the legend by different letter groups (a, b, c) for each aging scenario. GTL = gas‐to‐liquid; LTMO = low‐toxicity mineral oil.

The 14‐d acute toxicity study on earthworms demonstrated that earthworm survival was significantly affected by different NABF treatments (Figure 1). All 3 types of soil aging treatments (i.e., fresh and 10 and 30 °C) exhibited significant differences in earthworm survival between NABF treatments (Supplemental Data, Table S1). Earthworms had consistently lower survival in the diesel treatment relative to the control group, with 0 worms surviving at day 14 for the freshly spiked soil (Figure 1A) and the spiked soil aged for 90 d at 10 °C (Figure 1B), and 16.7% of worms surviving at day 14 for the spiked soil aged for 90 d at 30 °C (Figure 1C). The LTMO treatment also had significantly lower earthworm survival relative to the control group for the freshly spiked soil (Figure 1A) and the spiked soil aged for 90 d at 10 °C (Figure 1B); however, for the spiked soil aged for 90 d at 30 °C, there was 100% survival and no difference from the control group (Figure 1C). Between the 2 GTL NABFs, GTL_10–22_ was less toxic to earthworms than GTL_11–24_ in the freshly spiked (Figure 1A) and in the spiked soil aged for 90 d at 30 °C (Figure 1C). The GTL_10–22_ treatment never had mortality greater than was observed in the control group. The GTL_11–24_ treatment had no difference in survival from the control group in the spiked soil aged for 90 d at 10 °C (Figure 1B), but had moderate toxicity in both the freshly spiked soil (Figure 1A) and the spiked soil aged for 90 d at 30 °C (Figure 1C; 16.7 and 33.3% survival in these soils, respectively).

### Plant germination studies

The alfalfa germination assay showed a high amount of germination in the presence of the 4 NABFs in sandy loam soil (Supplemental Data, Figure S2). For alfalfa, there were no differences in seed germination among control groups and the different NABF treatments, in freshly spiked soil or under either of the aging scenarios (Supplemental Data, Table S2). The thickspike wheatgrass germination assay yielded more variable results among treatment groups than the alfalfa germination assay (Supplemental Data, Table S3). Germination in control and vehicle control soil was >80% or more germination for all 3 aging scenarios. In the freshly spiked soil scenario, germination rates were significantly lower in the 2 GTL treatments relative to the 2 control treatments, although the 2 GTL treatments did have higher germination rates than the diesel and LTMO treatments. In the soils aged for 90 d at 10 °C, there were similar germination rates in the control treatment, the 2 GTL treatments, and the diesel treatment, significantly higher germination in the vehicle control treatment than the GTL_10–22_ treatment, and significantly lower germination in the LTMO treatment relative to all other treatments. There were no significant differences in wheatgrass seed germination among any treatments in the soils aged for 90 d at 30 °C (Supplemental Data, Table S3).

The fourwing saltbrush germination assay (Supplemental Data, Table S4) produced similar germination patterns to the wheatgrass germination assay, despite lower overall germination rates. In the freshly spiked soil scenario, germination rates were significantly lower in the GTL_11–24_, diesel, and LTMO treatments relative to the 2 control treatments, with intermediate germination rates in the GTL_10–22_ treatment. In the soils aged for 90 d at 10 °C, there was significantly lower germination in the GTL_10–22_ treatment than in the vehicle control treatment, but neither were different from the germination rates in the control treatment. There was also significantly lower germination in the diesel and LTMO treatments relative to the control, vehicle control, and GTL_10–22_ treatments, with intermediate germination rates in the GTL_11–24_ treatment. There were no significant differences in saltbrush seed germination among any treatments in the soils aged for 90 d at 30 °C. Overall, for all 3 plant species, aging at 30 °C led to no significant differences between any of the NABF treatments and controls.

### Microbial community analysis

**Figure 2:**
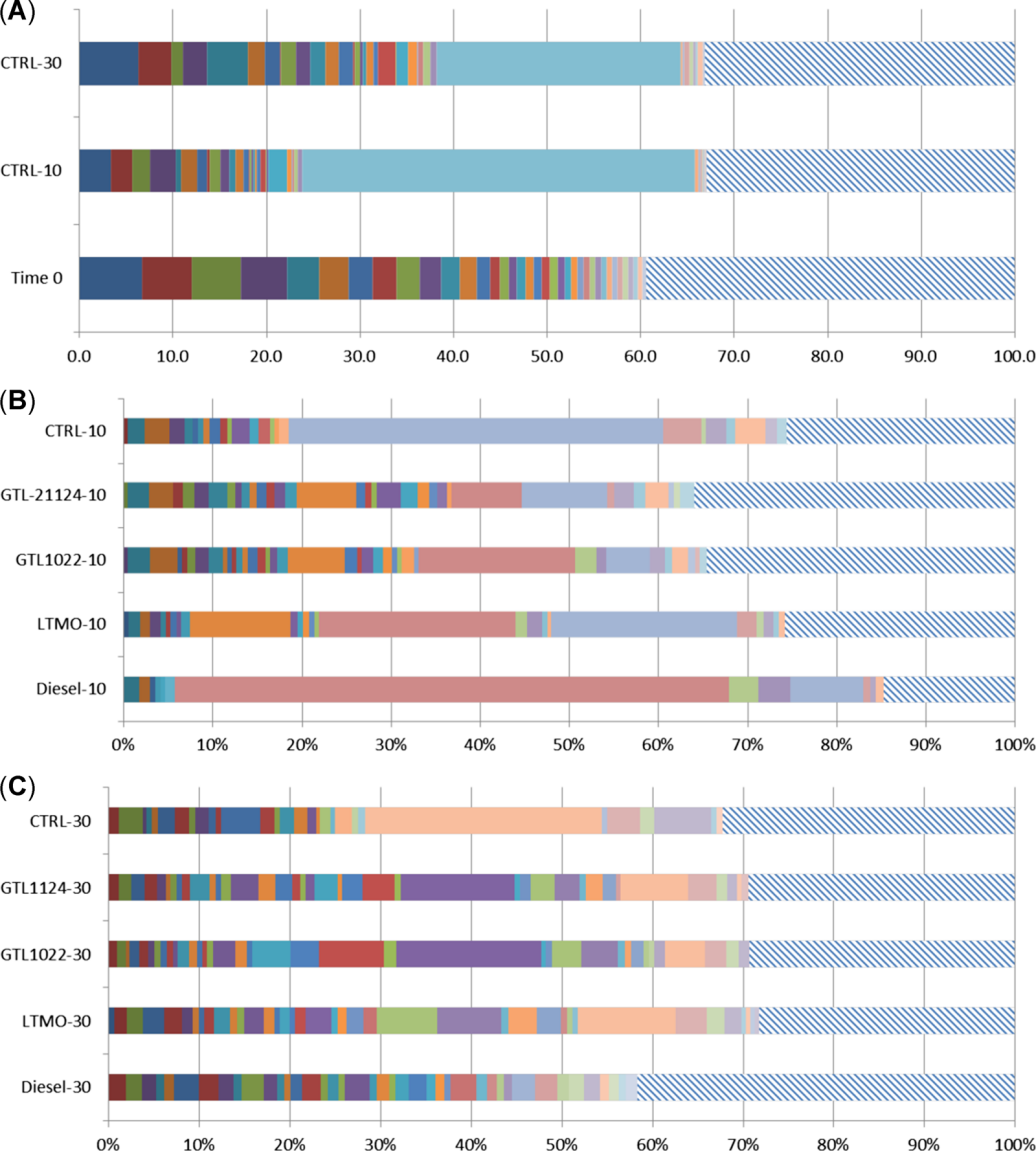
Microbial community in sandy loam soil incubated at different temperatures and nonaqueous base fluid treatment relative to untreated soil at time 0. Each species representing 0.5% of the total community at time 0 is represented as a colored bar. The hatched areas represent the remaining community. (**A**) Community diversity of controls by temperature relative to time 0. (**B**) Dominant community operational taxonomic units (OTUs) representing >0.5% by treatment after 90 d. of aging at 10 °C. (**C**) Dominant community OTUs representing more than 0.5% by treatment after 90 d. of aging at 30 °C. GTL = gas‐to‐liquid; LTMO = low‐toxicity mineral oil.

The exact composition of the community underwent a distinct shift or decomposition in species representation at different incubation temperatures as well as exposure to different NABFs. The sandy loam exhibited higher effective microbial diversity (eH′) at 30 °C incubations than 10 °C in the controls and diesel and LTMO treatments, but the opposite trend was seen in the GTL_10–22_ and GTL_11–24_ treatments, with only slightly higher effective microbial diversity at 10 than at 30 °C incubations (Table 2). Although overall species richness (*S*) of the controls was not affected by the incubation temperature, decomposition of the microbial community was apparent, with a 60% change in species identity over the 90‐d incubation period. Community shifts toward the order Actinomycetales were dramatic in all 90‐d incubations (Figure 2). Similarly, the Shannon–Weiner index (H′) showed a decreasing trend when warm climate sandy loam was incubated at 10 °C, in controls and when treated with diesel and LTMO, but a slight increase in the index was seen with the GTL treatments. Diesel‐treated soil resulted in the most severe declines in effective number of species, with a 74% decline in effective microbial diversity at 10 °C (Table 2).

**Table 2:** Common indices of biodiversity (richness, Gini–Simpson, Shannon–Weiner, and Evenness) and the true diversity (effective number of species) listed for each microbial community by soil type and treatment

Soil	Treatment		Richness (*S*)	Simpson (1‐D)	Shannon (H′)	Evenness (E)	Effective no. of species (eH′)
Sandy loam	Time 0		446	1	5	21.7	142
	Control	10°	418	1	3.5	5.3	31.8
	Control	30°	419	1	4.2	10.5	63.2
	Diesel	10°	309	1	2.1	1.5	8.4
	Diesel	30°	387	1	4.6	17.1	102.1
	LTMO	10°	194	1	3.6	6	36.5
	LTMO	30°	388	1	4.5	14.4	86
	GTL_10–22_	10°	458	1	4.5	15.1	92.3
	GTL_10–22_	30°	433	1	4.4	13.3	81
	GTL_11–24_	10°	492	1	4.8	18.7	116.1
	GTL_11–24_	30°	409	1	4.5	15	90.1

LTMO = low‐toxicity mineral oil; GTL = gas‐to‐liquid.

Sandy loam controls (no NABFs) at time 0 exhibited an average of 446 purported unique species or operational taxonomic units (OTUs), and 37 OTUs dominated by contributing at least 0.5% of the community. Half (50%) of the community mass was composed of 20 OTUs, but no OTU contributed >7% to the whole community. The same soil aged for 90 d at 10 °C had 419 OTUs, 25 of them contributing at least 0.5%; however, only 3 OTUs made up 50% of the community by mass, and 1 OTU contributed 42% of the entire microbial community. After 90 d of incubation at 30 °C, 420 OTUs were identified, with 30 dominant OTUs contributing at least 0.5%, 9 OTUs making up 50% of the community by mass, and 1 OTU representing 26% of the community. However, as seen in Figure 2A, 97% of the species identified as significant contributors (>0.5%) in the community at 30 °C made up a similar proportion of the community at time 0.

Treatments with NABFs at 10 °C resulted in distinct community decomposition shifts to ≥50% of the microbial community composition occurring as a single genus or microbial family. *Rhodococcus g*. dominated in all treatments, although the GTLs also contained a high relative abundance of *Nocardia g*. (Figure 2B). Remarkably, at 10 °C, the diesel‐treated community was 78% dominated by *Rhodococcus g*. At 30 °C, the sandy loam community was dominated by *Nocardia g.* in all treatments except the control (Figure 2C).

Analysis by principle components of NABFs and temperature (Supplemental Data, Figure S3) showed that the GTLs at the lower temperatures of 10 °C imposed the least amount of stresses on soil microbial communities’ phylogeny and relative abundance relative to untreated controls and the time 0 control than either LTMO or diesel NABFs. At higher temperatures of 30 °C, both GTLs resulted in similar soil microbial communities even though LTMO and diesel NABFs also generated large diverse communities at higher temperatures.

### Total hydrocarbon concentrations

With one exception, NABFs incubated in sandy loam soil at 10 °C showed small decreases in concentration during the 90‐d aging period. Loss of TPH was minimal for GTL_11–24_, and LTMO had the greatest loss at this temperature, followed by GTL_10–22_. Modeled (first‐order) dissipation half‐lives for the test substances were 75, 179, 48, and 148 d for GTL_10–22_, GTL_11–24_, LTMO, and diesel, respectively. Surprisingly, GTL_10–22_ and diesel incubated in sandy loam soil at 30 °C showed lower losses than at 10 °C. Apparent loss of TPH was minimal for diesel. Modeled (first‐order) dissipation half‐lives for the test substances were 152, 188, 57, and 314 d for GTL_10–22_, GTL_11–24_, LTMO, and diesel, respectively.

## DISCUSSION

In standardized toxicological testing, GTL products have been shown to exert less severe effects than their petroleum‐derived equivalents, consistent with our results. Terrestrial toxicity tests of related GTL products showed no toxicity in chronic and acute tests with earthworms (*E. fetida*), and an earthworm reproduction test on a GTL NABF with a very similar carbon range had a 56‐d median effect concentration (EC50) of 4160 mg/kg dry soil (Whale et al. 2018). Seedling emergence tests with soybean (*Glycine max*), tomato (*Lycopersicon esculentum*), mustard (*Sinapis alba*), and oats (*Avena sativa)* showed EC50 >1000 mg/kg dry soil for related GTL products; however, the perennial ryegrass (*Lolium perenne*) had a 560 mg/kg no‐observable‐effect concentration for growth. With a GTL NABF, soybean and tomato had an EC50 > 10 000 mg/kg, mustard had an EC50 of 4300 mg/kg, and oat and perennial ryegrass had an EC50 of 6200 mg/kg. Overall, GTL NABFs did not elicit strong toxicological responses in any of the standardized studies performed previously (Whale et al. 2018).

The present study examined 2 different GTL NABF products and their relative terrestrial toxicity in comparison with petroleum‐derived NABF products. Toxicity was assessed using an invertebrate survival study and seed germination studies. Although there was some variation for each study, overall at least one of the GTL NABFs was among the least toxic in each assay. Of the 2 GTLs, sandy loam soils spiked with GTL_10–22_ had less toxicity than those spiked with GTL_11–24_. This was illustrated by the earthworm 14‐d acute toxicity study, alfalfa germination and biomass study, and saltbrush germination study.

Survival, germination, and growth in GTL‐treated soils were very similar to survival in the controls. However, GTL_10–22_ freshly spiked soils did produce a significant toxic response in the germination of thickspike wheatgrass. In this same assay, the GTL_10–22_ aged soils did not have significant toxicity to thickspike wheatgrass. In fact, aged GTL_10–22_ soils did not result in toxicity in all assays performed. Therefore, it was concluded that in the case of the GTL_10–22_ NABF, terrestrial toxicity, which was already low by comparison, would continue to decline over time with aging, similar to observations on petroleum hydrocarbons (recently reviewed by Khan et al. 2019). In our particular study, the aging of the material did not correlate with loss of TPH. However, the biodegradability of hydrocarbons is well known, and several publications have looked at GTL fluids specifically, especially for their bioremediation potential (Rastegarzadeh et al. 2006; Getliff et al. 2012).

The soil used in the present study was assumed to be a healthy, productive soil containing a diverse microbial community. The dramatic microbial composition shift toward *Acetomycetes* seen in aged soil at all temperatures may be largely explained by the experimental conditions, under which moisture levels were maintained at field capacity (~26%). This moisture level may not mimic the semiarid environment of this native Texas soil, which would not normally maintain a level of moisture to support high populations of filamentous bacteria. Studies have shown that semiarid soils in the continental United States at circumneutral soil pH (pH 7–8.5) have some of the highest levels of soil microbial diversity compared with other soils in North and South America with much higher plant diversity but lower soil pH (Lauber et al. 2009).

Different native microbial populations thrive at different temperatures, and this can affect soil functionality, and/or temperature can alter the metabolic rates of the microbes present in the soil. Diesel treatment at 10 °C resulted in the most severe decrease in microbial community diversity, with *Rhodococcus* g. representing >79% of the total microbial population. *Rhodococcus g.* have been shown to metabolize environmental pollutants such as fossil fuels, toluene, naphthalene, and polychlorinated biphenyls and tend to be favored at low temperatures and other environments with low energy flux and fluctuating nutritional conditions; this may be explained by their very efficient management of nutrient requirements based on the flexibility of their metabolism and diversity of metabolic reactions (Whyte et al. 1998; Alvarez 2010).

At the higher 30 °C incubation temperature, treatment with drilling fluids caused a microbial community shift toward *Nocardia g*. but otherwise did not appear to greatly impact loss of diversity. The GTL_10–22_ treatment resulted in conditions most favorable to *Nocardia* spp., followed by the GTL_11–24_, and LTMO, treatments. *Nocardia g*. have been shown to prefer warmer temperatures and soils rich in organic matter. They are able to grow on grease, oil fats, xylenes, and alkylbenzenes and are commonly isolated from soils using a paraffin bait (Kurup and Schmitt 1971; Rodriguez‐Nava et al. 2007). It is possible that the chemical composition of the aged test materials changed based on incubation temperature, and this can affect both the apparent toxicity and the shift in microbial community. The slight reduction in species richness and diversity at 30 °C from that seen at 10 °C for GTL_10–22_‐ and GTL_11–24_‐treated soils is likely still a net functional benefit by favoring an increase in numbers and activities of known hydrocarbon degraders. It was concluded that GTL_11–24_ would be a better alternative for the environment than LTMO and possibly other petroleum‐derived oils, especially with freshly spiked soils. This is consistent with the previously published ecotoxicological information on GTL products (Whale et al. 2018).

For most of the assays in the present study, toxicity was highest for LTMO and diesel in soil. As expected, this toxicity was greater in freshly spiked soils versus soils in which the chemicals were aged. Of these 2 NABFs derived from petroleum, we found diesel most toxic to earthworms and seed germination, consistent with studies by Gainer et al. (2018, 2019b). It was the least refined product and contained more aromatics and sulfur than any other NABFs. Other studies have also identified diesel as having a high toxicity and being more toxic than other oils like mineral oil (Bennett 1984; Visser et al. 2002). It was observed that diesel had a secondary effect of drying soils at a more rapid rate. However, the acute toxicity assays were inspected daily so that the moisture content of all soils could be optimized for invertebrate survival and seed germination.

The LTMO treatment yielded surprising results in the earthworm assay. It was lethal to 100% of individuals in freshly spiked soils and soils aged at 10 °C for 90 d. In LTMO‐spiked soils that were aged at 30 °C for 90 d, all individuals survived (100% survival). In reviewing other assays, we found that LTMO‐spiked soil aged at 30 °C for 90 d also produced a high amount of germination (alfalfa and wheatgrass). Although there was some effect on seed germination with LTMO soils freshly spiked or those aged at 10 °C for 90 d (wheatgrass and saltbrush), the effect was not as drastic as with the earthworm study.

One reason for the drastic difference in earthworm survival could be the changes in the native microbial populations due to temperature (30 vs 10 °C) that we observed. Another reason that may have played a part in low earthworm toxicity was dissipation (volatilization and biodegradation) of the lighter hydrocarbons in the NABFs during the aging period (supported by our analytical data). The hydrocarbon content of LTMO was limited to shorter chains (C_11_–C_15_), which are more likely to volatilize. During the aging process, the soil containers were opened, soils were mixed regularly, and moisture was added. It is likely that a significant percentage of the LTMO dissipated in those 90 d of aging (supported by our analytical data), making that soil less toxic. Therefore, it was concluded that LTMO is highly toxic initially in terrestrial environments, but given the right conditions its toxicity dissipates over time.

It is possible that the incubated soils dried out in between the periodic moisture adjustments and that we did not adjust moisture frequently enough to compensate. Microbial activity could be severely depressed by the lack of moisture, leading to less degradation. Alternatively, the combination of temperature and treatment may have produced a microbial community with little capacity for hydrocarbon degradation.

We have made similar observations as those described above for NABFs in a silt loam soil (34% sand, 54% silt, 12% clay; 2.5% organic carbon; pH = 7.0; Hughes et al. 2013). Specifically, in freshly spiked soil, GTL_11–24_ had the most benign soil ecotoxicity profile, driven largely by its lack of earthworm and plant (alfalfa, saltbrush) toxicity. In comparison with sandy loam, GTL_10–22_ had a slightly higher toxicity profile in freshly spiked silt loam soil. The most toxic base oils tested were LTMO and diesel. We observed no large differences in persistence among the NABFs in silt loam soil; half‐lives for all products were slightly higher in soils incubated at 10 than at 30 °C, but overall none of the products would be considered persistent in silt loam soil. Despite no real differences in persistence, there were differences in the soil ecotoxicity profiles in the aged soils.

## CONCLUSIONS

Our study has bridged gaps in GTL knowledge by investigating the terrestrial toxicity of 2 GTL NABFs in comparison with other standard petroleum NABFs. Overall, NABFs aged in soil had more benign toxicity profiles than in freshly spiked soils. Incubation at 30 °C produced the greatest overall reduction in the invertebrate and plant toxicity for all the NABFs tested, but increased adverse effects on the soil microbial community were seen (as reflected in lower Shannon's diversity indices). Both diesel and LTMO had higher soil ecotoxicity profiles than the 2 GTL products in soils aged at 10 °C. This was driven largely by toxicity to invertebrates. In soils aged at 30 °C, diesel had the highest ecotoxicity profile, primarily because of slight toxicity to saltbrush germination and a reduction in microbial diversity. The 2 GTL products and LTMO had similar ecotoxicity profiles in soils aged at 30 °C.

## Supplemental Data

The Supplemental Data are available on the Wiley Online Library at DOI: http://10.1002/etc.4658.

## Supporting information

This article includes online‐only Supplemental Data.

Supporting information

## Data Availability

The raw data are available from the corresponding author on request (todd.anderson@ttu.edu).
