# Supplementary material for: Terrestrial Toxicity of Synthetic Gas‐to‐Liquid versus Crude Oil–Derived Drilling Fluids in Soil
Source: Environ Toxicol Chem. 2020 Feb 11;39(3):721–30. doi: 10.1002/etc.4658 (PMC7065218; doi:10.1002/etc.4658)
Supplement: Supplementary file 1 — Supporting information [file ETC-39-721-s001.docx]

Supplemental Information for Manuscript:

*Terrestrial toxicity of synthetic gas-to-liquid (GTL) versus crude oil-derived drilling fluids in soil*

**Table S1:** Sample size (N), significance level (α), statistical power and p-values of log-rank tests for differences in earthworm survival times between each treatment pair and each aging scenario. Statistically significant p-values are denoted by asterisks.

**Table S2**: Numerator and denominator degrees of freedom (*u, v*), F-statistic, significance level (α), statistical power and p-values of one-way randomization ANOVAs among alfalfa seed germination rate treatments for each aging scenario.

**Table S3:** Numerator and denominator degrees of freedom (*u, v*), F-statistic, significance level (α), statistical power and p-values of one-way randomization ANOVAs among wheatgrass seed germination rate treatments for each aging scenario. Statistically significant p-values are denoted by asterisks.

**Table S4:** Numerator and denominator degrees of freedom (*u, v*), F-statistic, significance level (α), statistical power and p-values of one-way randomization ANOVAs among fourwing saltbrush seed germination rate treatments for each aging scenario. Statistically significant p-values are denoted by asterisks.






B

A

**
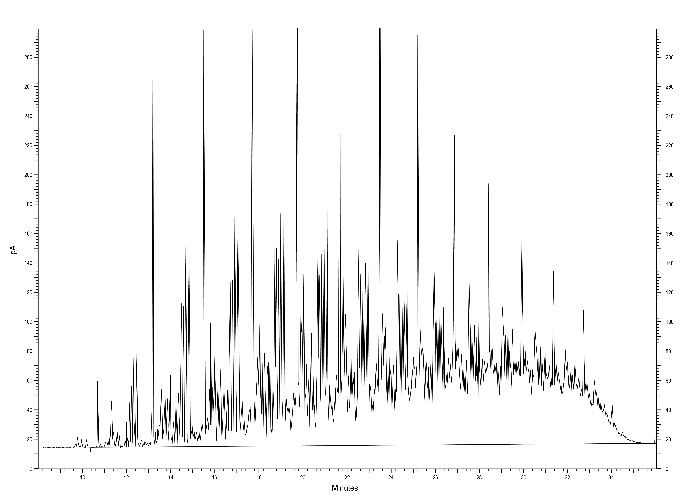

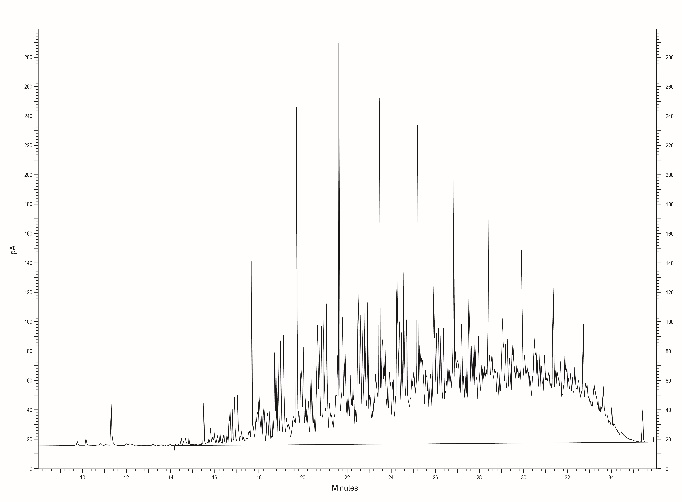
**

C

D

**Figure S1**: GCxGC chromatograms of NABFs. Panel A is GTL_10-22_. Panel B is GTL_10-22_ after incubation in sandy loam soil for 90 d at 30° C. Panel C is GTL_11-24_. Panel D is GTL_11-24_ after incubation in sandy loam soil for 90 d at 30° C.

B)

C)

**Figure S2:** Proportion of seeds germinated in each replicate of non-aqueous base fluid (NABF) treatment and aging scenario for A) alfalfa (*Medicago stavia*); B) wheatgrass (*Elymus lanceolatus*); and C) saltbrush (*Atriplex canescens*). Proportion germinated was calculated after 14 d in alfalfa and wheatgrass and after 21 d in saltbrush. Statistically significant differences between treatments are denoted by different letter groups (A, B, C) for each aging scenario. Control (v) is a vehicle control treatment of only a methanol spike used for dosing the NABF treatments.


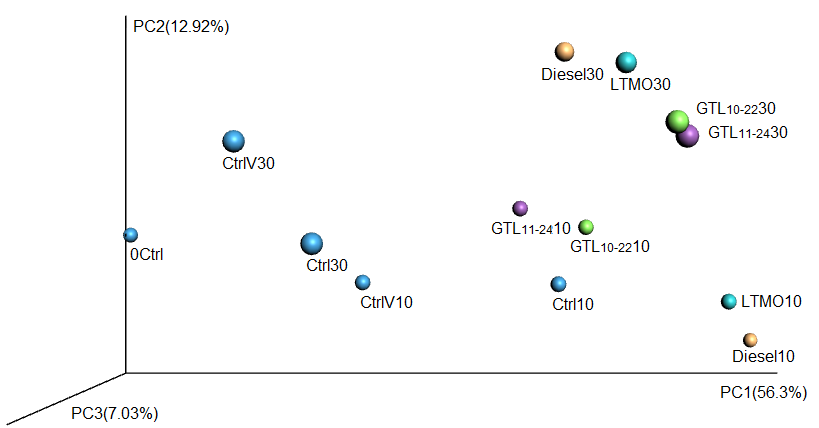


**Figure S3**. Principle coordinants analysis of weighted UniFrac distances. Beta-diversity implies that microbial communities shifts with different chemical compounds in the first axis and separated by temperature in the second axis.
